# Long Noncoding RNAs and Mitochondrial Homeostasis in the Development of Diabetic Retinopathy

**DOI:** 10.3389/fendo.2022.915031

**Published:** 2022-06-06

**Authors:** Renu A. Kowluru

**Affiliations:** Department of Ophthalmology, Visual and Anatomical Sciences, Wayne State University, Detroit, MI, United States

**Keywords:** Diabetic retinopathy, long noncoding RNAs, mitochondria, epigenetics, diabetes

## Abstract

Retinopathy is one of the most devastating complications of diabetes, which a patient fears the most. Hyperglycemic environment results in many structural, functional, molecular and biochemical abnormalities in the retina, and overproduction of mitochondrial superoxide, induced by hyperglycemic milieu, is considered to play a central role in the development of diabetic retinopathy. Expression of many genes associated with maintaining mitochondrial homeostasis is also altered. Recent research has shown that several long noncoding RNAs, RNAs with more than 200 nucleotides but without any reading frames, are aberrantly expressed in diabetes, and altered expression of these long noncoding RNAs is now being implicated in the development of diabetes and its complications including retinopathy. This review focuses the role of long noncoding RNAs in the development of diabetic retinopathy, with a special emphasis on the maintenance of mitochondrial homeostasis.

## Introduction

Incidence of diabetes is increasing at an alarming rate around the world; 151 million people had diabetes in 2000, this number is now 463 million adults, and is expected to pass 780 million by 2045 (1). Diabetes accounts for 6.7 million deaths in 2021-one death every 5 seconds, and this devastating disease does not know any social, economic or ethnic boundaries. Elevated blood glucose in the circulation, either due to impaired insulin production or resistance to properly use it, results in damaging small and large blood vessel in the body, resulting in many micro- and macro-vascular complications including nephropathy (kidney), neuropathy (nerve), retinopathy (retina, back of the eye) and cardiomyopathy (heart) (2). Over time, chronic high blood glucose affects almost every organ in the body, one in three adults with diabetes present chronic kidney disease, one in two peripheral nerve abnormalities and over 80% patients show some signs of retinopathy (3). Thus, diabetes is a whole-body chronic disease, and is commonly called as a ‘disease of its complications’.

## Long Noncoding RNAs

**Figure 1:**
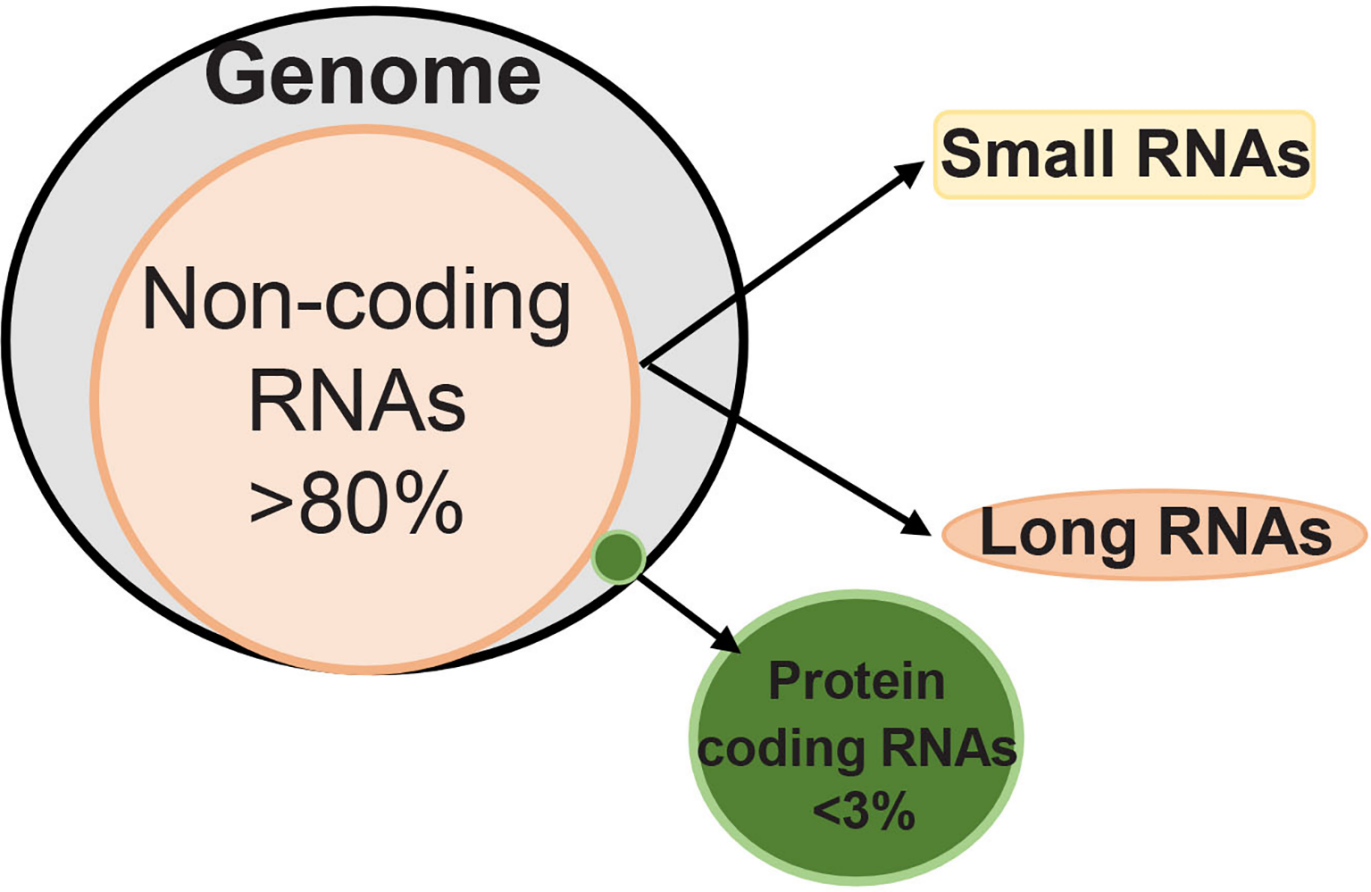
Human genome has less than 3% RNAs that can code for proteins, and over 80% of RNAs are noncoding. Depending on the length of their transcripts, noncoding RNAs are classified as ‘small’ and ‘long’ noncoding RNAs. While small noncoding RNAs have less than 200 nucleotides, long noncoding RNAs have over 200 nucleotides.

Technological development in transcriptome-wide sequencing has now documented that although majority of the mammalian genome is actively transcribed into RNA, only 2-3% is further translated into protein (4). Thus, most of the DNA does not code for the proteins, and this ‘non-coding DNA’ has a heterogeneous group of noncoding RNAs including transfer RNA, circular RNA, micro RNA (miRNA, less than 20 base pairs) and long noncoding RNA (LncRNAs, more than 200 base pairs) (**Figure 1**). The exact number of non-coding RNAs in the human genome is still not clear, but recent transcriptomic/bioinformatic studies have put their numbers in the tens of thousands (5). These noncoding RNAs, however, are not totally unfunctional, they can be alternatively spliced and/or processed into smaller products, and may have a hidden layer of internal signals (especially those derived from introns) that could control transcription, chromatin architecture etc. Noncoding RNAs are implicated in numerous cellular processes including cell cycle and metabolism (6–8). Thus far more than 2000 miRNAs and 30,000 LncRNAs have been discovered in humans, and these noncoding RNAs are responsible for regulating one third of the genes in the genome (9, 10). Although both miRNAs and LncRNAs RNAs are transcribed by RNAs polymerase II, miRNAs are better conserved than LncRNAs, and while miRNAs regulate mRNA at transcriptional and post-transcriptional levels, LncRNAs can remodel chromatin and genomic imprinting and regulate gene expression through sequence complementarity with RNAs or DNAs (11, 12). LncRNAs have four-fold higher tissue specificity compared to miRNAs (13, 14). LncRNAs are mRNA-like transcripts, but they do not possess any stable open reading frames, and compared to mRNAs, expression levels of LncRNAs are typically lower (14). Furthermore, while mRNAs have to be translated into proteins for carrying out specific cellular functions, LncRNAs are the functional unit and can function in different subcellular compartments based on local molecular interactions, and stronger tissue-specificity (15).

**Figure 2:**
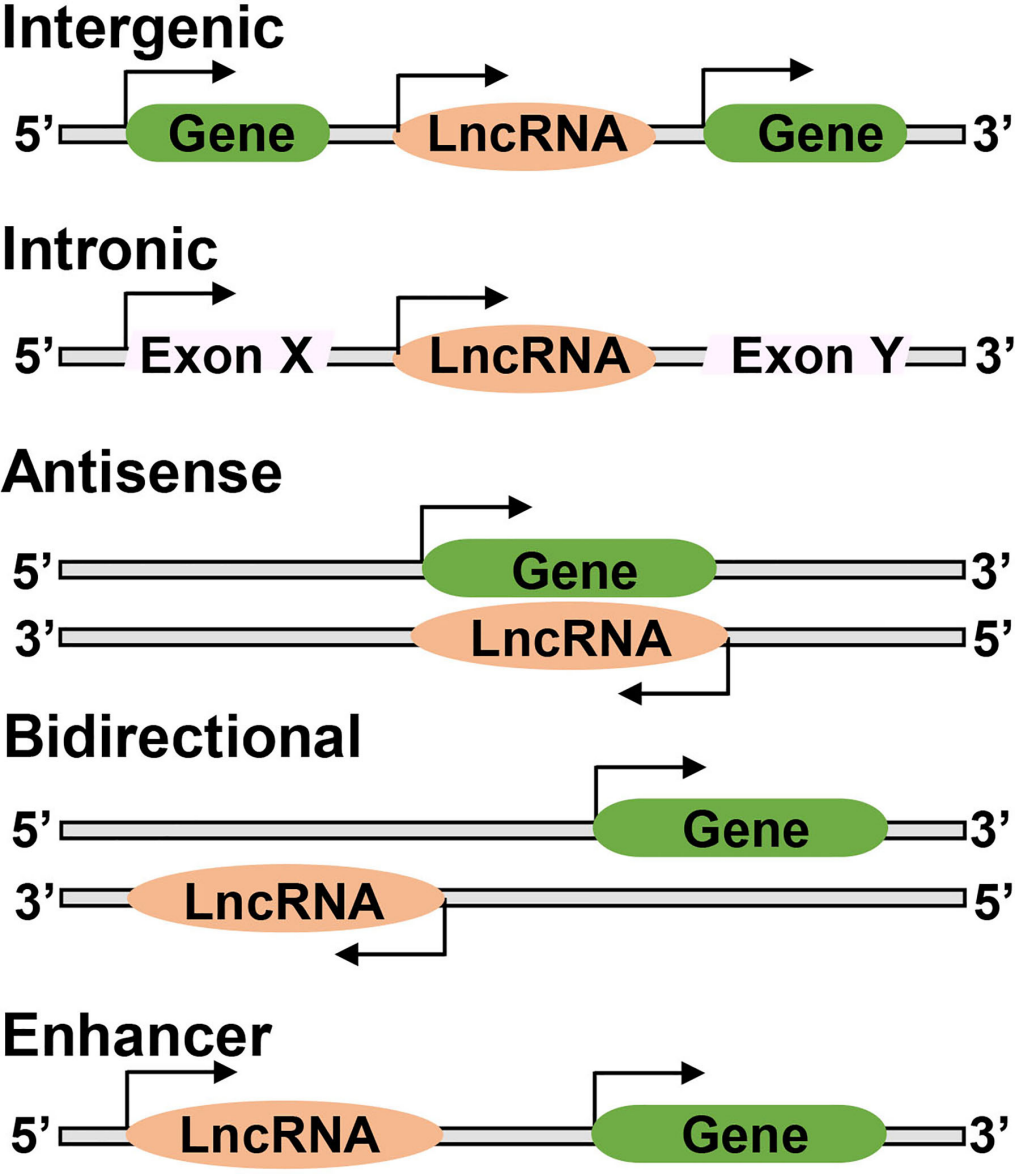
Long noncoding RNAs may have different genomic locations; between two protein coding genes ‘Intergenic’, or located within an intron of a coding gene ‘Intronic’, or overlap with one or more introns and exons of a different transcript on the same or opposite strand, respectively ‘Antisense’, or be within 100 kb of a coding transcript of the opposite strand ‘Bidirectional’, or present in the enhancer regions close to the promoter ‘Enhancer’.

Based on their genomic location, LncRNAs are classified into various subclasses, ‘intergenic LncRNAs’ located between two protein coding-genes without intersecting with any protein-coding genes, ‘intronic LncRNAs’ transcribed from intronic region of a protein coding-gene and overlap with protein-coding genes, ‘antisense LncRNAs’ transcribed from complementary strand of either protein coding or non-protein coding genes, ‘bidirectional LncRNA’ initiating in a divergent fashion from a promoter of protein-coding gene, and ‘enhancer LncRNA’ synthesized at the enhancers (**Figure 2**). LncRNAs act as a guide *via* interacting with the modifying complexes or transcription factors, directing them to specific genes or loci, or they may function as a scaffold by binding its multiple effector partners at the same time with proteins. LncRNAs also act as ‘decoy’ by binding to the transcription factors or miRNAs, repressing gene expression, or as an ‘enhancer’ by changing chromatin architecture to recruit transcription factors and promote transcription (16). They bind to DNA or RNA in a sequence-specific manner, or can act as sponges for miRNAs or as scaffolds to provide stability, and facilitate transcription factor binding (7, 17, 18). Thus, LncRNAs are capable of interacting with RNA and DNA.

LncRNAs are mainly encoded by nuclear genome and are overall more numerous in the nucleus, a significant number is also present in the cytoplasm, where they are more stable than their nuclear counterparts (19–21). LncRNAs modulate chromatin interactions in the nucleus, and generally help in signal transduction and posttranscriptional control of gene expression in the cytoplasm (22). In addition to nuclear genome, ~15% of the human mitochondrial transcriptome is made of noncoding RNAs, including three LncRNAs- LncRNA *ND5*, LncRNA *ND6* and LncRNA *CytB*. Mitochondrial LncRNAs are located in the regions of the mtDNA complementary to the genes that encode for *ND5*, *ND6 of* complex I and* cytochrome* B of complex III* *respectively, and these mitochondrial genome encoded LncRNAs primarily form intermolecular duplexes with their respective complementary mRNAs (23, 24). However, these mitochondrial genome-encoded LncRNAs are not restricted to the mitochondria, instead, they can operate in the nucleus (24). Nuclear genome-encoded LncRNAs can also move from the nucleus to the mitochondria and the mitochondrial genome-encoded LncRNAs can be aberrantly transported to the nucleus, and this aberrant shuttling of crosstalk of LncRNAs is associated with abnormalities in energy metabolism (25). Accumulating research indicates that LncRNAs play important roles in diverse biological processes, and are identified as key regulators in pathological processes in a range of diseases including cancer, diabetes and Alzheimer (26–29), and their role in diabetic macro- and microvascular complications is an emerging area of research (30).

## Diabetic Retinopathy

High circulating glucose leads to increased risk of many eye problems, including increase in intraocular pressure and damage to the lens and the retina. Damage to the retinal microvasculature, commonly known as diabetic retinopathy, is the leading cause of acquired blindness in the working age adults in developing countries. In the initial stages of this progressing disease, patients may be asymptomatic, but their retinal microvasculature begins to become fragile, and as the duration of diabetes increases, retinopathy also progresses and the damaged retinal vasculature begins to leak. With time, lack of oxygen in the microvasculature leads to neovascularization, and ultimately, to vision loss (31, 32). Early diagnosis and treatment options of diabetic retinopathy, however, are limited, and it is critical to identify effective methods of its diagnosis and therapeutic targets to retard progression of this sight-threatening disease. In the first five years of diabetes, retinopathy is rare in type 1 diabetic patients, but after 20 years of diabetes, over 80% of patients develop retinopathy. In type 2 diabetic patients, 21% of patients present early signs of retinopathy at the time of their diagnosis of diabetes, and as with type 1 diabetic patients, most type 2 diabetic patients also develop some degree of retinopathy over time. In 2020, over 100 million diabetic adults had retinopathy, and among those, 28.5 million had vision-threatening retinopathy, and with increase in the incidence of diabetes, these numbers are expected to increase to over 160 million and 45 million, respectively in 2045 (33). Although pathological evidence is mainly seen in the retinal microvasculature, research has shown that nonvascular cells of the retina are also affected; they experience functional and structural alterations including retinal ganglion cell apoptosis, loss of contrast sensitivity and alterations in the electroretinogram (34, 35). Normal cell-cell interactions are also affected leading to the blood-retinal barrier breakdown and neuronal cell dysfunction (6, 36). In addition, Optical Coherence Tomography has also shown ganglion cell loss in the retina of diabetic patients with or without retinopathy, however, diabetic patients with moderate or severe retinopathy show more thinning of their ganglion cell-inner plexiform layers compared to patients without any retinopathy (37).

### Impaired Mitochondrial Homeostasis

Diabetic retinopathy is a multifactorial disease with a complex etiology, and increase in oxidative stress-mitochondrial damage is considered to play a major role in the development of this blinding disease (2, 38–42). Retina is rich in polyunsaturated fatty acids and has a high oxygen uptake (43), making it a good target of increased oxidative stress in diabetes. In diabetes, increased cytosolic ROS, generated by activation of Ras-related C3 botulinum toxin substrate 1 (Rac1)-NADPH oxidase 2 (Nox2)-activation of gelatinase matrix metalloproteinases in diabetes damage mitochondria in retinal capillaries (44, 45). In addition, due to high circulating glucose, electron flux through the electron transport chain system is increased, and the complex III becomes dysfunctional, resulting in increased accumulation of ROS (40, 46). Mitochondrial structural and functional stability is impaired including increased production of superoxide radicals and damage to their membrane potential. Cytochrome C leaks out from the mitochondria in the cytosol, which accelerates capillary cell apoptosis, a phenomenon which is followed by the histopathology characteristic of retinopathy (38, 41, 47, 48). Mitochondria have their own DNA (mtDNA), which encodes genes for 13 proteins that are essential for the electron transport chain functioning; diabetes damages mtDNA and reduces the gene transcripts of mitochondrial genome-encoded *ND1* and *ND6* of complex I, and *cytochrome B* of complex III are reduced. In addition, mitochondrial dynamics is imbalanced, while fusion is decreased, fission is increased, and the removal of damaged mitochondria in impaired. Impairment in mitochondrial homeostasis in diabetes is further exacerbated by the suboptimal biogenesis of mtDNA (47, 49–52). Thus, instability in mitochondrial structure, function and genome plays a central role in the development of diabetic retinopathy.

Aldose reductase, an enzyme with a high ‘Km’ for glucose, using NADPH as a cofactor, converts glucose to sorbitol. This reduces availability of NADPH for glutathione biosynthesis, increasing oxidative stress. In diabetes, polyol pathway is activated in the retina and its capillary cells (53, 54). Diabetes increases non-enzymatic glycation of amino acids in proteins, lipids and nucleic acids, and this ultimately leads to advanced glycation end products (AGEs) (55); retinal capillaries have increased AGEs and their receptors (RAGEs), and AGEs-RAGEs are implicated in increased inflammation and capillary cell loss in diabetic retinopathy (56). Diacyl glycerol-protein kinase C cascade is also activated by high glucose, and protein kinase C activation in retinal vasculature is associated with increased vascular permeability, alterations in blood flow and stimulation of neovascularization seen in diabetic retinopathy (57). Furthermore, activation of protein kinase C, polyol pathway and increased AGEs accumulation can also increase oxidative stress, and can also be initiated by hyperglycemia-induced overproduction of mitochondrial superoxide, further strengthening the role of mitochondrial damage in diabetic retinopathy (40, 58). In addition, many inflammatory markers including cytokines and leukostasis, are also elevated in diabetic retinopathy (59), and retinal glial cells, the cells that are between vasculature and neurons of the retina, also initiate the inflammatory cascade (60).

Hyperglycemia is the main instigator, but systemic factors, such as high blood pressure, cholesterol and smoking are also closely associated with the development and progression of diabetic retinopathy (61), and also in the exacerbated and accelerated mitochondrial damage (62). However, multifactorial nature and complex etiology of diabetic retinopathy makes it difficult to identify a link between any specific metabolic abnormality and the development of this progressive blinding disease.

### Genetics and Diabetic Retinopathy

Genetic factors appear to play some role in diabetic retinopathy, but the exact role of genetic contribution in the development/progression of diabetic retinopathy has remained unclear. Significant association between the variations in the polyol pathway gene encoding aldo-keto reductase family 1-member B1, *AKR1B1*gene, has been documented by a meta-analysis (63). Type 2 diabetic patients in Pakistan with Pro12Ala polymorphism are at a reduced risk of developing diabetic retinopathy (64). Jackson Heart Study has shown an association between P-selectin and diabetic retinopathy (65). Another genome-wide association study (GWAS) has shown association with a zinc finger protein associated with transcriptional regulation, ZNF600. Polymorphisms at the regulatory regions of some of the genes including *VEGF* and endothelial nitric oxide synthase and paraoxonase1, have been considered as risk alleles for the susceptibility or progression of diabetic retinopathy (66). Genotyping of single nucleotide polymorphism has identified new loci on chromosome 6 associated with diabetic retinopathy, but none of the variants have shown any statistical significance (67). A recent GWAS meta-analyses of type 2 diabetic patients have shown enrichment of variants in the genes involved lipid catabolism and transport, oxidative stress and cell degeneration pathways and the risk of diabetic retinopathy (68). In addition, an association for the z-2 microsatellite and rs759853 on aldo-keto reductase family 1 member B, and some evidence for polymorphisms in *VEGF*, intercellular adhesion molecule 1 genes have also been documented by systemic meta-analyses, but a follow up of the analysis has not reported any loci after adjustment for multiple (69). A study of cohort from China with proliferative diabetic retinopathy has identified *INSR* gene as a possible susceptibility candidate for severe diabetic retinopathy, but this study is underpowered (70). Siblings of affected individuals has been shown to have three-fold higher risk of severe diabetic retinopathy, but diabetic patients with similar risk factors do not show the same range in the severity of retinopathy, when evaluated by Early Treatment Diabetic Retinopathy Study Grading System (71). Thus, despite recent technological advancement in the genetic field, multifactorial pathogenesis of this blinding disease has made it difficult to identify definite genetic associations. In addition, variations in the severity of hyperglycemia and other systemic factors such as hyperlipidemia and blood pressure in the diabetic population have further compounded the problem.

## Epigenetics Modifications

Gene function is expressed in the form of protein, and changes in the DNA sequence can alter how it is transcribed into RNA, which eventually is coded for a particular protein. The expression of a gene is also affected by external factors, such as environment and lifestyle, and these ‘epigenetic’ changes can turn a gene ‘on’ and ‘off’ without altering the DNA sequence. Although epigenetic processes are essential to many organism functions, their up- or down-regulation can have major adverse health and behavioral effects; they are now implicated in many diseases including cancer, cardiovascular diseases and diabetes and its complications. Some of the major epigenetic modifications include methylation of cytosine (DNA methylation), methylation and acetylation of histones and noncoding RNAs (72). Methylation of the 5′ position of the cytosine forms 5-methyl cytosine (5mC), and this process is facilitated by DNA methyl transferases (Dnmts) and condenses the chromatin, both genomic DNA and mitochondrial DNA undergo methylation. However, dioxygenases-ten-eleven translocation (Tets) can rapidly convert 5mC to hydroxymethylated to 5-hydroxymethyl cytosine (5hmC), resulting in transcriptional activation (73–75). Lysine in the histone proteins can be acetylated, relaxing the chromatin structure to allow transcription factors binding, and this is modulated by a balance between acetyl transferring and removing enzymes, histone acetyltransferases and histone deacetylases, respectively (76–78). Lysine or arginine residues in a histone can also be modified by the addition of one, two, or three methyl groups, and methylation status of histones is also maintained by opposing enzymes, addition of methyl group by histone methyltransferases and removal of methyl group by demethylases. Unlike histone acetylation, gene repression or activation is determined by the site of methylation and the number of methyl groups (79, 80). Moreover, epigenetics modifications have potential to modify the gene expression independently, or one modification can lead to the other, to ultimately regulate gene expression (75, 81). Noncoding RNAs may not be the main epigenetic components, but they can also regulate gene expression without altering the DNA sequence.

### Epigenetics Modifications and Mitochondrial Damage

The role of epigenetic modifications in many chronic diseases, such as cancer and Alzheimer disease, is being investigated for several years, and their role in diabetes and its complications is now emerging (82–85). In diabetes, Dnmts and Tets are activated in the retina and its vasculature, and dynamic DNA methylation-hydroxymethylation at the promoters of *Rac1* and *matrix metalloproteinase 9* is associated with their activation and mitochondrial damage. Furthermore, DNA at the promoter of polymerase gamma, a gene associated with mtDNA biogenesis, and at *MutLH1* (associated with mtDNA damage repair) is hypermethylated, further damaging the mitochondria. Mitochondrial DNA itself is also hypermethylated in diabetes, which impairs its transcription resulting in a compromised electron transport chain system (41, 86, 87). Thus, epigenetics plays a vital role in maintaining mitochondrial homeostasis in diabetic retinopathy.

LncRNAs are now considered as a new class of epigenetic regulators because they can regulate epigenetic modification by modulating histone or DNA modifications, and can also act as a scaffold that interacts with epigenetic enzymes or methylation and acetylation complexes. LncRNAs can regulate histone methylation and acetylation simultaneously, subsequently regulating the gene expression (88). Thus, LncRNAs can epigenetically modify the expression of a gene without altering its DNA sequence.

## Long Noncoding RNAs and Diabetic Retinopathy

Leading scientists in the field have reviewed how LncRNAs play a role in the development of diabetic retinopathy, focusing mainly on inflammation and angiogenesis, but the field is still in its infancy. Many laboratories are now focusing on identifying LncRNAs associated with various metabolic and functional abnormalities implicated in the development of diabetic retinopathy (89–94). LncRNA profiling performed in the retina of streptozotocin-induced diabetic mice has shown over 300 LncRNAs that are aberrantly expressed at two months of diabetes, with 214 showing downregulation and 89 upregulation (95), and profiling in the fibrovascular membranes of patients with diabetic retinopathy have identified 427 differentially expressed LncRNAs, 263 upregulated and 164 downregulated (96). Both of these profiling studies have identified differentially expressed LncRNAs that are enriched in inflammatory and angiogenic pathways including Metastasis-associated lung adenocarcinoma transcript 1 (MALAT1), Hypoxia-inducible factor 1 HIF-1 and tumor necrosis factor alpha.

In diabetic retinopathy, Lnc*MALAT1*, which is one of the most highly expressed and conserved LncRNAs, is upregulated in the *in vivo* and *in vitro* models, and also in the circulation of the patients with diabetic retinopathy (97). Aqueous humor and fibrovascular membranes of diabetic patients also have increased Lnc*MALAT1* (95). Inhibition of Lnc*MALAT1* protects retinal photoreceptors, and alleviate diabetic neurodegeneration (98). Although Lnc*MALAT1* is implicated in normal physiologic functions such as vascular growth (99), its elevation in diabetes is shown to act both as pro-inflammatory and apoptotic (100–102). *MALAT1* sequence has NF-*k*B transcription factor binding sites, and NF-*k*B is intimately associated with the induction of proinflammatory cytokines; in diabetic retinopathy, NF-*k*B is activated and many proinflammatory cytokines are upregulated (59, 103). Upregulation of cytokines is shown to play a major role in the development of diabetic retinopathy; in fact, diabetic retinopathy is also considered as a low grade chronic inflammatory disease. In experimental models of diabetic retinopathy, Lnc*MALAT1* expression is augmented by hypoxia contributing to the proliferative response in endothelial cells (104). In retinal endothelial cells Lnc*MALAT1* inhibition ameliorates cell migration-angiogenesis, neurodegeneration and monocyte chemotactic protein-1, and also prevents inactivation of the master transcription factor, nuclear factor erythroid 2-related factor, Nrf2 (91, 98, 105). Our recent studies have documented an antioxidant defense role of Lnc*MALAT1* in diabetic retinopathy; *via* affecting the interactions of nuclear factor erythroid 2-related factor 2 (Nrf2) and its intracellular inhibitor Kelch-like ECH-associated protein 1, Lnc*MALAT1* upregulation suppresses transcriptional activity of Nrf2, and this impairs the transcription of antioxidant defense genes, such as heme oxygenase 1 and superoxide dismutase (91). Inhibition of LncRNA *MALAT1* alleviates diabetes-induced neurodegeneration (98). LncRNA, antisense RNA to INK4 locus (Lnc*ANRIL*) with binding sites for NF-*k*B, is also upregulated in diabetes, and is implicated tube formation-proliferation in endothelial cells *via* regulation of VEGF expression (106). Another LncRNA Myocardial infarction associated transcript (Lnc*MIAT*), is also regulated by NF-*k*B; high glucose increases the NF-*k*B and *MIAT* binding, further regulating inflammatory cytokines and apoptosis (107). LncRNA maternally expressed gene 3 (Lnc*MEG3*), which increases endoplasmic reticulum stress and inhibits cell proliferation (108), is considered to negatively correlate with VEGF, and in patients with diabetic retinopathy, while the serum levels of VEGF are upregulated, Lnc*MEG3* are downregulated (109). Another LncRNA HOX antisense intergenic RNA (Lnc*HOTAIR*) is upregulated in the vitreous of diabetic retinopathy patients and in retinal endothelial cells by high glucose, and its inhibition is shown to prevent increase in retinal vascular permeability and VEGF in diabetic rodents (110). Thus, the role of LncRNAs in inflammation, oxidative stress and angiogenesis is now at the forefront of ongoing research.

## Long Noncoding RNAs and Mitochondrial Homeostasis in Diabetic Retinopathy

Mitochondrial dysfunction plays a critical role in many diabetic complications, and as mentioned above, in diabetic retinopathy damaged mitochondria accelerate apoptosis of capillary cells, which precedes the development of histopathology (48, 111). Retinal mitochondrial homeostasis is impaired, they become swollen and their damaged membranes leak cytochrome C in the cytosol, superoxide levels are elevated, mitochondrial dynamics are disturbed, mtDNA is damaged and mitochondrial genome-encoded genes including *cytochrome B* is downregulated, and the complex III of the electron transport chain is inhibited (41, 47). LncRNAs, whether encoded by mitochondria or nuclear genome, are also implicated in mitochondrial homeostasis, nuclear genome encoded Lnc*MALAT1* is translocated in the mitochondria of hepatoma cells, where it is shown to regulate mitochondrial apoptosis and mitophagy (25), and Lnc*MEG3, via* mitochondrial pathway, is shown to induce renal cell carcinoma apoptosis (112). Mice with *MALAT1* knocked out have lower levels of ROS and protein carbonylation in hepatocyte and islets (113). Furthermore, Lnc*MALAT1* is also shown to interact with multiple loci on the mtDNA in hepatoma cells (114). In addition, Lnc*HOTAIR* induces mitochondria-mediated apoptosis in head and neck squamous cell carcinoma *via* Bcl-2-BAX-Caspase-3 pathway (115), and cardiac apoptosis-related LncRNA (Lnc*CARL*) suppresses mitochondrial fission (116). In diabetic nephropathy, nuclear genome encoded LncRNA taurine-upregulated gene 1 is shown to modulate mitochondrial biogenesis by directly interacting with PGC1α (117), and depletion of Nuclear Enriched Abundant Transcript 1 (Lnc*NEAT1)* is shown to alter mitochondrial dynamics (118). This clearly shows that many nuclear genome-encoded LncRNAs have potential to regulate mitochondrial function.

**Figure 3:**
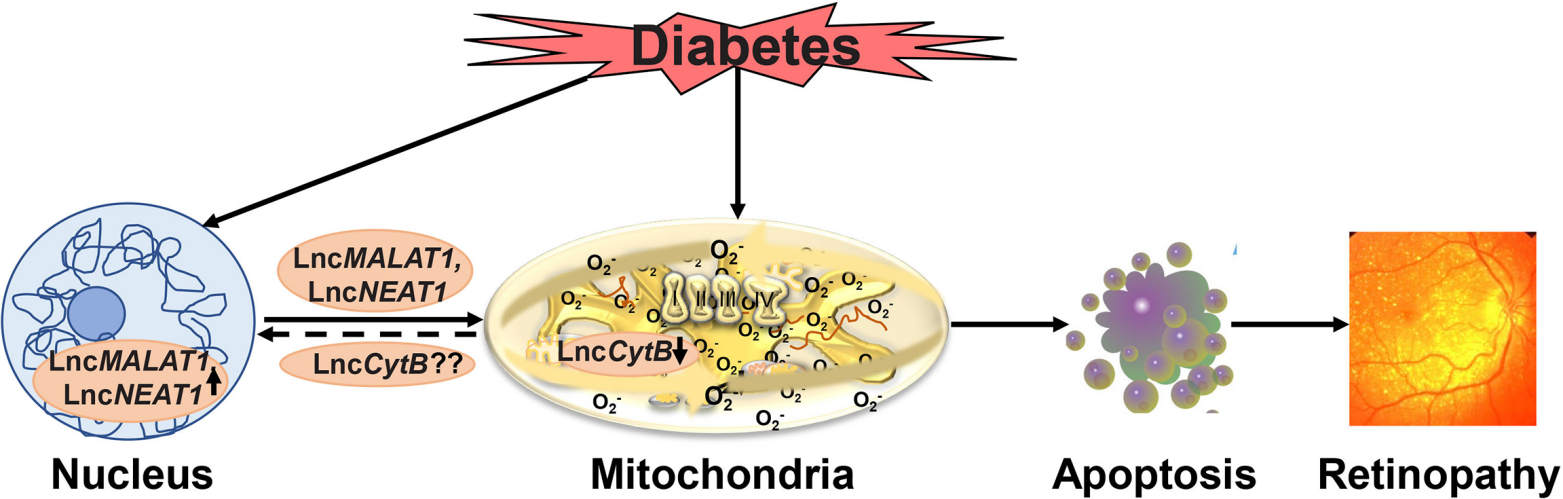
Hyperglycemic environment damages mitochondria and also elevates levels of various nuclear genome encoded LncRNAs including Lnc*MALAT1* and Lnc*NEAT1.* These LncRNAs, in addition to affecting metabolic abnormalities associated with diabetic retinopathy including inflammation, also move inside the mitochondria, and damage mitochondrial structural, functional and genomic stability. Furthermore, possible decrease in the mitochondrial genome encoded Lnc*CytB* further damages the mitochondria, and the damaged mitochondria accelerates apoptosis, ultimately, resulting in the development of diabetic retinopathy.

Focusing on diabetic retinopathy, our recent work has suggested a role of nuclear genome encoded Lnc*MALAT1* and Lnc*NEAT1* in regulation of mitochondrial functions, we have demonstrated that high glucose increases their mitochondrial localization in retinal endothelial cells, and siRNA of Lnc*MALAT1* or Lnc*NEAT1* prevents increase in ROS and alterations in mitochondrial membrane potential. As mentioned earlier, diabetes damages retinal mtDNA and impairs the transcription of mitochondrial genome-encoded genes, important for the functioning of the electron transport chain, which results in a vicious cycle of free radicals (41, 42, 119). Lnc*MALAT1* is shown to interact with multiple loci on mtDNA in hepatoma cells (114), and regulation of Lnc*MALAT1* or Lnc*NEAT1* by their respective siRNAs in retinal endothelial cells, prevents glucose-induced mtDNA damage and decrease in its transcription (93). Although mtDNA does not contain histones, it has nucleoprotein complexes, the nucleoids, and these nucleoids provide a stable environment for replication and repair of mtDNA (120). In addition, nucleoids also help in controlling mitochondrial metabolism in response to cellular demands (121, 122). Regulation of Lnc*MALAT1* or Lnc*NEAT1* also protects glucose-induced decrease in mtDNA nucleoids in retinal endothelial cells, suggesting a close cross-talk between nuclear genome-encoded LncRNAs and mitochondrial stability *via* their role(s) in increasing the vulnerability of the histone-free mtDNA in hyperglycemic milieu (**Figure 3**).

As reported above, mtDNA itself also encodes three LncRNAs, two of them transcribed from its L strand, and one from the H strand, the regions of the mitochondrial genome that are complementary to the genes that encode *ND5*, *cytochrome B* and *ND6* mRNAs (23, 24). Lnc*ND5*, Lnc*ND6* and Lnc*CytB* are 58, 34 and 14% as abundant as their complementary coding *ND5*, *ND6*, and *cytochrome B* mRNAs, and these LncRNAs primarily form intermolecular duplexes with their respective complementary mRNAs, suggesting their functional role in either stabilizing or regulating their partner mRNAs (23, 24). Their role in metabolism/diseases, however, is still unclear. Our recent work (unpublished) has demonstrated decreased expression of Ln*CytB* in retinal endothelial cells in high glucose conditions, and shown its role in protecting mtDNA vulnerability to nuclease damage and *cytochrome B* expression; overexpression of Ln*CytB* ameliorates glucose-induced increase in mtDNA damage, decrease in mitochondrial genome-encoded nucleoids and decrease in *cytochrome B* expression. These studies have suggested that downregulation of Lnc*CytB* in the mitochondria in hyperglycemic milieu reduces the protective nucleoids, which makes mtDNA more susceptible to the damage. The damaged mtDNA compromises the electron transport chain and the vicious cycle of free radicals continues to self-propagate, thus implying an important role of this mitochondrial genome-encoded LncRNA in mitochondrial homeostasis in diabetic retinopathy. Although our initial results show significant decrease in Lnc*CytB*, we do not see any significant decrease in the other two mitochondrial genome-encoded LncRNAs-Lnc*ND5* or Lnc*ND6* in either *in vitro* or *in vivo* models of diabetic retinopathy. Despite significantly lower abundance of Lnc*CytB* is significantly lower compared to the other two mitochondrial genome-encoded LncRNAs, complex III is considered to play a major role in superoxide generation in diabetic retinopathy, and the activity of complex III, but not complex I, is downregulated (2, 38, 40)

As described above, nuclear genome-encoded LncRNAs can move inside the mitochondria and regulate mitochondrial homeostasis, and aberrant transport of mitochondrial genome-encoded Lnc*CytB* in the nucleus of hepatoma HepG2 cells as compared with normal hepatic HL7702 cells suggests that the aberrant shuttling of LncRNAs in mitochondria-nucleus crosstalk could be important for abnormal energy metabolism in malignant cells (25). Weather mitochondrial genome-encoded LncRNAs also translocate outside the mitochondria in diabetic retinopathy needs investigation.

The focus of this review thus far has been on how LncRNAs can directly affect mitochondrial homeostasis in diabetic retinopathy, but many epigenetic modifications including histone modifications and methylation of DNA of many genes associated with mitochondrial metabolism and mitochondrial DNA itself, also affect mitochondrial stability (47, 87). Long noncoding RNAs are considered to play a significant role in regulating histone modifications and DNA methylation, for example, Lnc*HOTAIR* is shown to interact with the histone modifying polycomb repressive complex II and histone demethylase 1 (LSD1) (18). In diabetic retinopathy, LSD1 is activated, and its recruitment at the promoter of mitochondrial damaging matrix metalloproteinase-9 is increased. Silencing of LSD1 prevents hyperglycemia-induced damage to mitochondrial structure and function in retinal endothelial cells, suggesting an important role of this enzyme in mitochondrial homeostasis in diabetic retinopathy (123). Furthermore, in hyperglycemic milieu, *MALAT1* silencing directly reduces Enhancer of Zeste homolog 2 (Ezh2), an enzyme which catalyzes dimethylation/trimethylation of histone 3 lysine 27 (90), and Ezh2 activation in diabetes helps recruit DNA methylation enzymes at the promoter of *matrix metalloproteinase-9*, activating its transcription (124). Also, role of LncRNAs in regulating methylation of mtDNA or DNA at the promoters of other genes important genes associated with mitochondrial homeostasis needs further investigation. LncRNAs can also act as microRNA sponges, and in retinal pigment epithelium cells exposed to high glucose, upregulation of miR-34a is reversed by upregulation of MEG3 and Sirtuin 1 (125); overexpression of Sirtuin1 prevents mitochondrial damage and the development of retinopathy in diabetic mice (126). Lnc*MALAT1*, *via* targeting mi125b, inhibits glucose-induced angiogenesis in retinal endothelial cells (127). As the list of miRNAs implicated in the development of diabetic retinopathy grows, their interactions with LncRNAs in diabetic retinopathy is an open area that would need further attention.

## Long Noncoding RNAs as Biomarkers and Therapeutic Options

Recent technical advances have allowed the LncRNA field to make great progress, the list of LncRNAs and their potential association with diseases is expanding rapidly, and their relatively stable nature in the body fluids, ease of obtaining body fluids and detecting them using commonly used molecular biology techniques makes them attractive biomarker candidates (128, 129). The results of a meta-analysis using data from over 1400 patients have shown that LncRNAs can serve as promising diagnostic biomarkers (130). For example, higher levels of LncRNA *LIPCAR* are associated with a higher risk of cardiovascular mortality (131), and LncRNA *NONHSAT112178* can be a possible predictive biomarkers for coronary artery disease (132), Lnc*HOTAIR* is highly expressed in saliva samples of oral squamous cells from carcinoma patients, making it a strong candidate for metastatic oral cancer diagnosis (133), and Lnc*MALAT1* is elevated in plasma and urine of prostate cancer patients (134). In diabetic retinopathy, Lnc*HOTAIR* and Lnc*MALAT1* have shown some discrimination between patients with nonproliferative and proliferative retinopathy (135) and plasma and aqueous humor levels of LncRNA small nucleolar RNA host gene 5 are negatively correlated with the development of diabetic macular edema (136). A recent study has shown a strong correlation between the differential expressions of nine LncRNAs including Lnc*ANRIL*, Lnc*HOTAIR* and Lnc*MIAT*, in the vitreous and serum samples of patients with or without diabetic retinopathy; these promising results suggest the possible use of serum LncRNAs as a potential biomarker of diabetic retinopathy, however, their validation in a larger cohort is needed (137). Thus, many LncRNA are now being implicated in regulating various pathways associated with the development of diabetic retinopathy, and future research and technical advancements may show us their role as biomarker or therapeutic target for its development, and/or its progression.

Like any new technology, LncRNA field also faces some challenges- the transcriptome studies suggest that ~80% of the genome is transcribed into RNA (138), but it is not clear how many of those LncRNAs are functional and what concentration of that LncRNA is required to achieve their biological effect. Also, LncRNAs can affect gene expression *via* many different mechanisms, it is difficult to predict how many different mechanisms one single LncRNA might use to control expression of a gene in a given disease. Fortunately, technical advances in genome editing and in surveying LncRNA molecular mechanisms have significantly advanced our ability to definitively characterize function of LncRNAs, making these molecules attractive targets for therapeutic intervention for diabetic retinopathy. LncRNAs in the mitochondria are also being investigated as potential blood-based biomarkers for cardiac remodeling in patients with hypertrophic cardiomyopathy (139), and with additional research in the field and technical advancements, their use in maintaining mitochondrial homeostasis in diabetic retinopathy is very optimistic, which will give diabetic patients hope of not losing their vision.

## Author Contributions

The author confirms being the sole contributor of this work and has approved it for publication.

## Funding

This article based on the work supported in part by grants from the National Institutes of Health (EY014370, EY017313, and EY022230) and from The Thomas Foundation to RK, and an unrestricted grant from Research to Prevent Blindness from the Department of Ophthalmology, Wayne State University.

## Conflict of Interest

The author declares that the research was conducted in the absence of any commercial or financial relationships that could be construed as a potential conflict of interest.

## Publisher’s Note

All claims expressed in this article are solely those of the authors and do not necessarily represent those of their affiliated organizations, or those of the publisher, the editors and the reviewers. Any product that may be evaluated in this article, or claim that may be made by its manufacturer, is not guaranteed or endorsed by the publisher.
